# Alteration and dysfunction of ion channels/transporters in a hypoxic microenvironment results in the development and progression of gastric cancer

**DOI:** 10.1007/s13402-021-00604-1

**Published:** 2021-04-15

**Authors:** Junling Chen, Minglin Zhang, Zhiyuan Ma, Dumin Yuan, Jiaxing Zhu, Biguang Tuo, Taolang Li, Xuemei Liu

**Affiliations:** 1 Department of Gastroenterology, Affiliated Hospital of Zunyi Medical University, Zunyi, 563003 Guizhou Province China; 2 Digestive Disease Institute of Guizhou Province, Zunyi, Guizhou Province China; 3 Department of Thyroid and Breast Surgery, Affiliated Hospital of Zunyi Medical University, Zunyi, 563003 Guizhou Province China

**Keywords:** Gastric cancer, Ion channel, Tumor microenvironment, Hypoxia, ROS, HIF

## Abstract

**Background:**

Gastric cancer (GC) is one of the most common malignant cancers in the world and has only few treatment options and, concomitantly, a poor prognosis. It is generally accepted now that the tumor microenvironment, particularly that under hypoxia, plays an important role in cancer development. Hypoxia can regulate the energy metabolism and malignancy of tumor cells by inducing or altering various important factors, such as oxidative stress, reactive oxygen species (ROS), hypoxia-inducible factors (HIFs), autophagy and acidosis. In addition, altered expression and/or dysfunction of ion channels/transporters (ICTs) have been encountered in a variety of human tumors, including GC, and to play an important role in the processes of tumor cell proliferation, migration, invasion and apoptosis. Increasing evidence indicates that ICTs are at least partly involved in interactions between cancer cells and their hypoxic microenvironment. Here, we provide an overview of the different ICTs that regulate or are regulated by hypoxia in GC.

**Conclusions and perspectives:**

Hypoxia is one of the major obstacles to cancer therapy. Regulating cellular responses and factors under hypoxia can inhibit GC. Similarly, altering the expression or activity of ICTs, such as the application of ion channel inhibitors, can slow down the growth and/or migration of GC cells. Since targeting the hypoxic microenvironment and/or ICTs may be a promising strategy for the treatment of GC, more attention should be paid to the interplay between ICTs and the development and progression of GC in such a microenvironment.

## Introduction

Gastric cancer (GC) is the fifth most common malignancy and the third most common cause of cancer-related death worldwide [1]. Due to the poor prognosis of GC, its related five-year survival rate does not exceed 30% in most countries [2, 3]. Therefore, intensive research on the pathogenesis of GC and the development of novel and effective drugs and treatment options are essential.

**Fig. 1:**
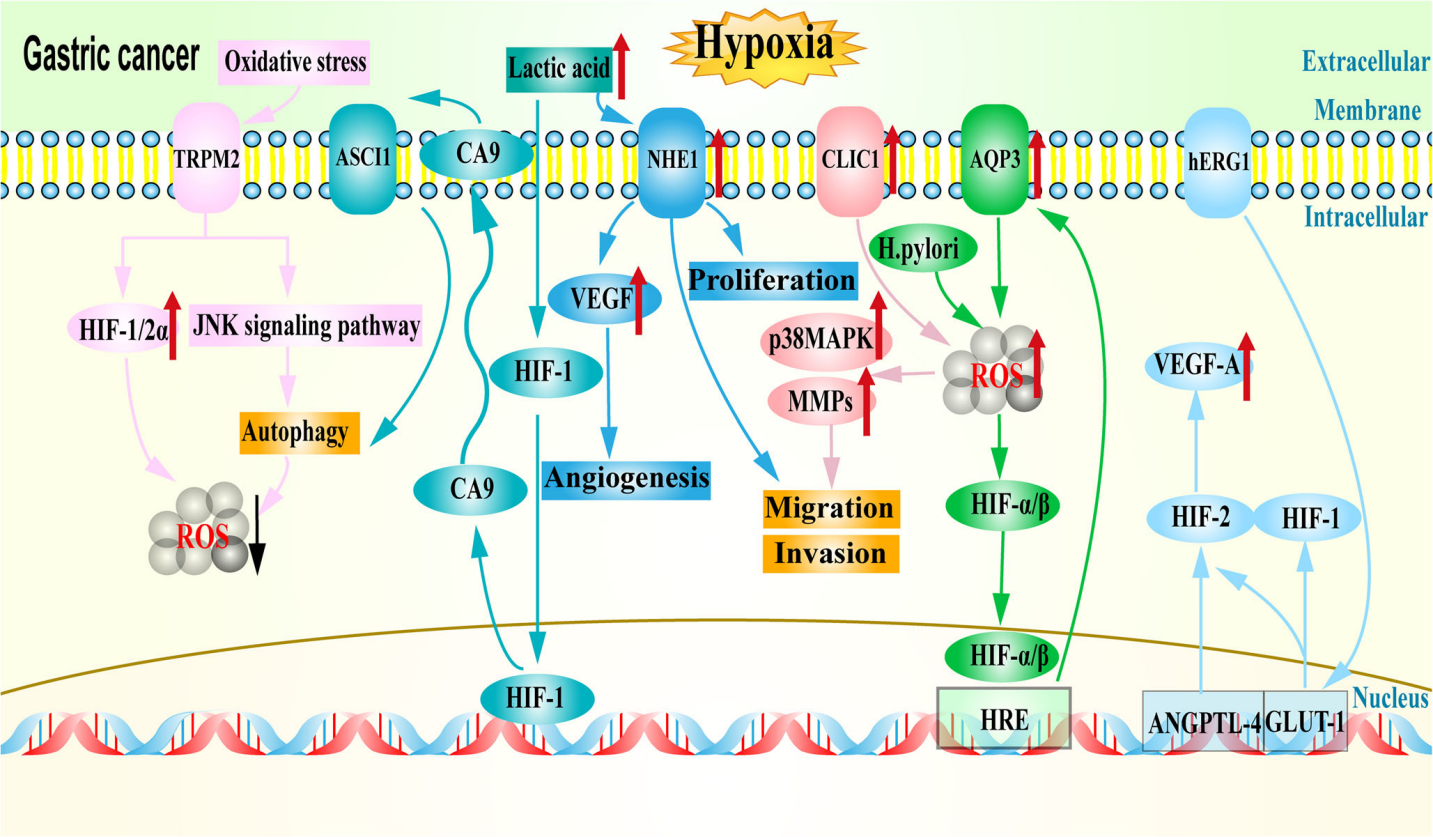
Diagram of ICTs in the hypoxic microenvironment regulating the development of GC. Different ICTs are shown in different colors; red arrows indicate upregulation and black arrow indicates downregulation

Recent studies have shown that cancer progression and metastasis depend on bidirectional interactions between cancer cells and their environment, which together form the tumor microenvironment (TME) [4]. The TME is a complex, dynamic network composed of cellular and noncellular components [5, 6] that is characterized by hypoxia, an acidic extracellular pH (pHe), high lactate levels, strongly elevated adenosine concentrations, low levels of glucose, ATP and nutrients and the presence of VEGF and many other cytokines and growth factors [7–9]. Among these factors, hypoxia is of particular concern. Solid tumors generally contain hypoxic regions that can trigger important cellular changes [10]. Moreover, cancer cells metabolize glucose in the form of glycolysis (‘Warburg effect’), and hypoxia can further aggravate the dependence on glycolytic fueling, which results in the production of large amounts of lactic acid [11, 12]. However, cancer cells maintain an intracellular pH (pHi) equal to or higher than that of normal cells, which indicates that these cells can increase the net acid excretion level [13–16]. This can be achieved by upregulating the expression and/or activity of acid excretion carriers and/or by downregulating the expression and/or activity of acid addition carriers. Therefore, the role of ion channels and transporters (ICTs) has become a focus of cancer research (Fig. 1).

ICTs are a class of membrane proteins [17] that are closely related to almost every biological process in the human body, such as cell proliferation, apoptosis, migration, volume regulation, epithelial secretion, contraction and regulation of smooth muscle cells, and regulation of the pH balance and cell cycle [18–22]. Moreover, increasing evidence indicates that ICTs may be involved in interactions between cancer cells and the TME. Recent studies have shown that hypoxia can simultaneously increase transient receptor almelastatin-like7 channel (TRPM7) expression and induce HIF-1α accumulation in androgen-independent prostate cancer cells. TRPM7 silencing, however, significantly promoted hypoxia-inducible factor 1 alpha (HIF-1α) degradation through the proteasome and inhibited EMT changes in androgen-independent prostate cancer cells under hypoxic conditions [23]. Numerous studies have shown that HIF-1α acts as a main transcription factor that mediates hypoxic responses and promotes the transcription of angiogenic factors such as VEGF, which leads to an increase in glycolysis through the inhibition of mitochondrial oxidative phosphorylation [24]. In contrast, HIF-1α stability may depend on high levels of reactive oxygen species (ROS) in hypoxic cancer cells [25, 26], which can activate the NF-κB, TNF-α and STAT3 signaling pathways in inflammatory cells and tumor cells to release a variety of inflammatory cytokines involved in changes in the TME [27]. Recent studies have found that HIF-1α promotes the proliferation, migration, invasion, angiogenesis and endothelial-mesenchymal transition (EMT) of GC cells [28]. The levels of ROS have been found to be significantly increased in patients with GC, and abnormally high levels of ROS to induce oxidative stress, which can damage the gastric mucosa and be an important factor in the development of GC [29]. In addition, hypoxia can induce autophagy and an acidic extracellular pH through intermediate components or modes, which are also associated with GC progression and chemoresistance [30–34].

In this context, deciphering the crosstalk between ICTs and various components in the TME during hypoxic responses of GC cells deserves special attention. Previously, the physiological and pathophysiological significance of gastric ICTs in GC have been summarized [35]. Here, we review the ICTs that are regulated by hypoxia or regulate the response to hypoxia in GC, including aquaporin 3 (AQP3), chloride intracellular channel 1 (CLIC1), hERG potassium channel, acid-sensing ion channel 1 (ASIC1), Na^+^/H^+^ exchanger1 (NHE1) and transient receptor potential melastatin 2 (TRPM2), which have not been discussed in detail before.

## Ion channels regulate the response to hypoxia

### AQP3 participates in the development of GC through the ROS–HIF-1α–AQP3–ROS loop

Aquaporins (AQPs) are members of a specialized superfamily of membrane integral proteins [36] that includes 13 different aquaporin (AQP0-AQP12) isoforms, which are widespread among microorganisms, plants and mammals [37, 38]. Structurally, each ~30-kDa AQP monomer consists of six transmembrane helical domains (H1 – H6) and two short helical segments (HB and HE). Four AQP monomers aggregate on the plasma membrane to form a tetramer [39, 40]. AQPs form the main transcellular route of water transport in the gastrointestinal tract and are essential for maintaining body water homeostasis and ensuring digestive and absorptive functions. Currently, at least 10 aquaporins (including AQP1–5 and AQP7–11) have been found to be expressed in the human stomach. Among these AQPs, AQP3, AQP4 and AQP5 present differential expression levels between GCs and corresponding normal tissues [41, 42]. Among the AQPs that have been investigated in GCs, AQP3 is the best studied and subject of this review. AQP3 localizes to the basal layers of epithelial cells and is a major player in barrier hydration and water and osmolyte homeostasis in the human body [43, 44].

A previous study showed that AQP3 is expressed in goblet cells and is positively correlated with gastric intestinal metaplasia (GIM) severity [45]. In addition, it has been found that AQP3 is highly expressed in GC tissues and regulates the proliferation, migration and invasion of human GC cells through a variety of signaling pathways [42]. The PI3K/AKT signaling pathway is, for example, involved in AQP3-mediated transport of glycerol, which can lead to the proliferation of GC cells [46]. A recent study revealed crosstalk between AQP3 and several components involved in the hypoxia response in *H. pylori*-infected gastric mucosa. Several studies have shown that *H. pylori* remains the most common bacterium under these conditions and that infection with this bacterium can lead to chronic gastritis and an increased risk of GC [47]. HIF-1α activation through persistent hypoxia is closely related to the invasive tumor phenotype and an unfavorable prognosis in patients with GC. In the gastric mucosa of *H. pylori* infected patients, high levels of ROS are produced by *H. pylori* itself or NADPH, and increased accumulation of intracellular ROS stabilizes HIF-1α bound to HIF-1β and increases the expression of HIF-1α in gastric epithelial cells. HIF-1α is a well-known inducer of VEGF, and the ROS-HIF-1α axis plays an important role in the production of VEGF in *H. pylori* infected gastric epithelial cells [48, 49]. The HIF-1 complex can activate the transcription of many target genes under hypoxic conditions, which results in adaptation to the hypoxic environment. It has been shown that the heterodimeric HIF complex can bind to HREs in the AQP3 promoter region and thereby activate the transcription and upregulation of AQP3. Increased AQP3 levels can mediate ROS uptake and accelerate the accumulation of ROS, and the ROS-HIF-1α-AQP3-ROS loop can further upregulate the expression of AQP3. Hypoxia is a characteristic of tumors and in the presence of persistent hypoxia, the ROS-HIF-1-AQP3-ROS loop may continue to operate, even in the absence of *H. pylori* [48]. Another study showed that AQP3 increases autophagy in GC cells and promotes the resistance of GC cells to cisplatin through autophagy, which suggests that AQP3-based therapy may be employed in future GC treatment strategies [50]. In summary, it has been found that AQP3 may be involved in the development of GC, and that crosstalk between AQP3 and the hypoxia response may be one of the underlying mechanisms.

### CLIC1 participates in the metastasis and invasion of GC cells by regulating hypoxia-reoxygenation-induced intracellular ROS

Jentsch and co-workers showed that chloride channels can be divided into six categories: (1) voltage-gated Cl^−^ channels (ClCs), (2) cystic fibrosis transmembrane conductance regulator (CFTR), a cAMP-activated Cl^−^ channel, (3) volume-sensitive osmolyte and anion channels (VSOACs), also referred to as swelling-activated Cl^−^ channels, (4) Ca^2+^-activated Cl^−^ channels (CACCs), (5) p64-related chloride intracellular channels (CLICs) and (6) γ-aminobutyric acid and glycine receptors, which represent ligand-gated Cl^−^ channels [51]. Among these channels, the CLIC family of proteins consists of seven different members: CLIC1, CLIC2, CLIC3, CLIC4, CLIC5, p64 and parchorin [52]. Following the successful isolation of the p64 protein and the subsequent cloning of its gene, the first human paralogue, NCC27 (later renamed CLIC1), was cloned from the myelomonocytic cell line U937. CLIC1 is located on human chromosome 6p21.3 and encodes a protein of 241 amino acids that is expressed in the nucleus, cytoplasm and cell membrane [53–55]. Ulmasov et al. found that CLIC1 is expressed in the apical domains of several types of simple columnar epithelia, including those of the glandular stomach, small intestine, colon, bile ducts, pancreatic ducts, airways, tail of the epididymis and renal proximal tubules [56]. Chen et al. also found a trace of CLIC1 expression in normal gastric tissue [57]. In addition, it was found that CLIC1 may physiologically participate in changes in cell volume, membrane potential regulation, acidification of intracellular organelles, proliferation and differentiation of cells and cell cycle progression [58].

Increasing evidence indicates that CLIC1 is associated with a variety of malignancies, including pleomorphic human sarcoma [59], glioblastoma [60], ovarian cancer [61–63], liver cancer [64], pancreatic cancer [65], gastric cancer [57, 66, 67] and colorectal cancer [53, 68, 69]. CLIC1 is highly expressed in GC and is strongly correlated with lymphatic invasion, lymph node metastasis, perineural invasion and pathological stage. In addition, it has been found that high CLIC1 expression may inhibit the proliferation of GC cells and promote their apoptosis, migration and invasion [57, 67]. It has been shown that CLIC1 acts as a sensor and effector during oxidative stress [70] and affects the progression of various tumors through ROS regulation [69–73]. Previous studies have also shown that hypoxia-reoxygenation (H-R) may increase intracellular ROS levels to activate the MAPK/ERK pathway and that the CLIC1 protein participates in the migration of LOVO colon cancer cells by regulating the ROS/ERK pathway during H-R [69]. Similar to its role in colon cancer, CLIC1 is involved in the migration and invasion of GC cells by regulating intracellular ROS production. At has amply been shown that ROS might act not only to impair cellular and protein function, but also as second messengers in cellular processes involving changes in cellular redox status, such as migration, differentiation and replication. Cellular oxidation can activate kinases such as mitogen-activated protein kinase (MAPK), protein kinase C (PKC) and protein kinase B (PKB), and it has been found that changes in cellular redox status are characteristic of some pathological conditions, particularly chronic inflammatory conditions, tumor states and degenerative processes [70]. Under H-R conditions, the level of ROS in SGC-7901 GC cells was found to be increased, and the expression of p-p38, MMP-2 and MMP-9 to be significantly increased. These processes are regulated by MAPK signal transduction pathways [71, 74, 75]. Functional inhibition of CLIC1 downregulates H-R-induced intracellular ROS production in GC cells, and the p38 MAPK inhibitor Sb203580 inhibits H-R-induced GC cell motility. These results suggest that CLIC1 may regulate the migration and invasion of GC cells through the ROS-mediated p38 MAPK signaling pathway [71]. In conclusion, a clear correlation between CLIC1 expression and the migration and invasion of GC cells has been observed, and the subsequent finding that CLIC1 regulates the response to hypoxia during the development of GC contributes to our understanding of the mechanism underlying GC cell invasion and metastasis.

### hERG1 channels induce GC and regulate VEGF-A secretion via HIFs

Potassium channels represent the largest group of ion channels in the human genome. Based on similarities in amino acid sequences and functional properties, the genes encoding pore-forming subunits can be subdivided into three major families: (1) channel subunits with six or seven transmembrane domains and one pore loop (6/7TM-1P), which are known as voltage-gated and Ca^2+^-activated K^+^ channels, (2) channel subunits with four transmembrane domains and two pore loops (4TM-2P), which are known as K2P channels and (3) channel subunits with two transmembrane domains and one pore loop (2TM-1P), which are considered inward rectifying K^+^ channels [76]. Potassium channels are located on the outer cell membrane and participate in fundamental processes such as cell membrane excitability, ion and solute transport and cell volume regulation. Potassium channels are physiologically involved in maintaining the external K^+^ balance in epithelial cells of the gastrointestinal system and play an important role in the production of gastric acid and the secretion of gastric juice [77, 78]. The ether-a-go-go (EAG) potassium channel family, which encompasses alpha subunits of six-transmembrane-domain voltage-gated K^+^ (VGK) channels, has been divided into three subfamilies: EAG, EAG-related gene (ERG) and EAG-like (ELK) K^+^ channels [79]. The human EAG-related gene (HERG) was cloned using a human hippocampal cDNA library, is located at chromosome 7q35–36, and encodes 1159 amino acids [80]. hERG potassium channels are normally expressed in heart, vascular smooth muscle, brain, thymus and adrenal gland, but not in the normal gastric mucosal epithelium [81, 82].

Up till now, research on hERG channels has focused on the role of this potassium channel in cardiac repolarization and long QT syndrome (LQTS) [82]. However, these channels have also been found to be overexpressed in a wide range of human cancers [83–85], and it has been reported that the activity of hERG affects three major functions related to tumor cell biology: proliferation, invasion and tumor angiogenesis [86]. hERG mRNA and protein are expressed specifically in GC cells, and the hERG protein localizes to the cytoplasm and membrane of GC cells [87]. hERG1 expression is related to the Lauren intestinal type GC, its fundus localization, grading, TNM stage, lymph node involvement, serosal and venous invasion and VEGF-A expression [86–88]. It has been shown that hERG1 channels are strongly regulated by hypoxia [89]. Under hypoxic conditions, the accumulation of HIF-1 directly upregulates VEGF expression and cooperates with a variety of other factors participating in angiogenesis [90]. Similar to findings in colorectal cancer, hERG1 channels regulate VEGF-A secretion in GC through Akt-dependent regulation of HIF (mainly HIF-2) transcriptional activity. In contrast, hERG1 inhibition decreases the expression of HIF-1α and HIF-2α-coregulated (GLUT-1) and HIF-2α-regulated (ANGPTL-4) genes without affecting the expression of the LDHA gene. Additionally, it has been found that a hERG1 inhibitor can decrease Akt activity, and that PI3K/AKT inhibitor treatment can inhibit HIF-1 activity and significantly decrease VEGF-A secretion. Notably, hERG1 is expressed early during GC progression and may, therefore, be of value in predicting the prognosis, clinical course and/or response to chemotherapy in patients with GC [86, 88]. Considering that hERG1 is closely related to hypoxia and that HIFs remain promising anti-angiogenic agents and direct targets for interfering with the energetics of cancer cells to regulate their growth [91], the possibility of combining hERG channel blockers with drugs targeting hypoxia-related components or developing analogous schemes should be considered in future studies.

## Hypoxia affects the expression and function of ICTs in GC

### Hypoxia promotes the opening of ASIC1 channels, which regulate GC through autophagy

Acid-sensitive ion channels (ASICs), also known as H^+^-gated cation channels, are a class of ligand-gated cation channels belonging to the mechanosensitive epithelial Na^+^ channel/degenerin (ENaC/DEG) superfamily. ASICs are sensitive to amiloride and independent of voltage [92–94]. To date, five mammalian genes (Accn1–5) encoding seven ASIC subtypes (ASCI1a, ASIC1b, ASIC2a, ASIC2b, ASIC3, ASIC4 and ASIC5) have been cloned [95]. ASICs occur as trimers [96], and each ASIC subunit is composed of approximately 500 amino acids and consists of two hydrophobic transmembrane domains, i.e., transmembrane domain 1 (TMD1) and transmembrane domain 2 (TMD2), a large cysteine-rich extracellular loop and an intracellular domain containing the carboxyl (C) and amino (N) ends [97]. ASICs are almost ubiquitously expressed in the mammalian nervous system in both peripheral and central nerves, are involved in neurosensory mechano-transduction in multiple tissues and organs, and have been implicated in touch, pain, digestive function, baroreceptors, blood volume control and hearing [95, 97]. There is ample evidence indicating that ASICs play an important role in mucosal homeostasis of the upper gastrointestinal tract (stomach, esophagus and duodenum). To avoid tissue damage, the secretion of gastric acid must be strictly controlled according to the body’s needs, and acid-sensitive protective mechanisms must be present in all parts of the intestine that may be exposed to excessive lumen acid [98]. Currently, there is much scientific evidence supporting the notion that ASICs are expressed in a variety of cancers, play a role in the acidic microenvironment by regulating multiple malignant processes in tumors, including proliferation, invasion and migration, and can affect cell cycle progression [99–106].

It has been shown that cancer is a malignant disease characterized by microenvironmental hypoxia due to abnormal blood vessels and a poor blood flow. Under hypoxia, cells convert aerobic respiration into glycolysis, which results in the intracellular or extracellular accumulation of lactate and acidification of the extracellular environment. If the extracellular environment is acidified, HIF-1 is activated and binds to DNA to promote the expression of carbonic anhydrase 9 (CA9). CA9 on the cell membrane can alleviate intracellular acidosis via anion exchangers, which results in reduction in extracellular pH and the opening of ASICs [107]. ASIC1 participates in acidosis-mediated signal transduction through calcium influx, which is one of the mechanisms through which a low extracellular pH in the microenvironment promotes the growth and metastasis of tumors [100, 108–111]. To date, few studies have investigated the involvement of ASICs in the occurrence and development of GC. Zhang Q et al. found that autophagy plays an important role in the regulation of GC cell growth by ASIC1. Previous studies have shown that the acidic tumor microenvironment can induce the expression of autophagy-related genes and promote autophagy, and that autophagy represents the lysosomal catabolism pathway of proteins and organelles. It has also been reported that the RNA and protein expression levels of autophagy-related 5 (ATG5) and ASIC1 are increased in GC tissues. ASIC1 regulates autophagy through ATG5 activation. In a murine GC xenograft model, ASIC1 or ATG5 gene knockdown inhibited the growth of the tumor cells, whereas ASIC1 shRNA treatment led to decreased tumor volumes and prolonged survival times of the animals. Therefore, it was concluded that downregulation of ASIC1 inhibits the growth of GC by reducing autophagy [112]. Subsequent studies have shown that the expression of ASIC1 exhibits a significant correlation with an increased risk of GC as well as with GC cell migration and invasion [113, 114]. Thus, hypoxia causes acidification of the extracellular environment, which in turn promotes the opening of the ASIC1 channel and ultimately regulates GC through autophagy. ASIC1 inhibitors may be used as potential therapeutic drugs.

### Hypoxia can activate and upregulate NHE1, which is involved in the proliferation, migration and invasion of GC cells

Human Na^+^/H^+^ exchangers (NHEs) are encoded by the SLC9 gene family classified by solute carriers of transporters, including SLC9A1–9 (NHE1–9), SLC9B1–2 (NHA1, NHA2) and SLC9C1–2. Among them, the human NHE1 protein is encoded by SLC9A1 and contains a hydrophobic N-terminal membrane domain responsible for the transport of NHEs and a hydrophilic, intracellular long C-terminus necessary for NHE1 regulation [115]. NHE1 controls cell volume and pH, but is also involved in complex biological processes such as cell adhesion, migration, proliferation and mechano-sensation [116]. NHE1 is strongly expressed in the gastric mucosa and is one of the predominant isoforms in mucous cells that regulate the pHi, particularly in the presence of high gastric acid levels, which is important for gastric barrier action to acids and for maintaining a near-neutral pHi in the gastric mucosa [117]. In addition, NHE1 may be responsible for the initiation of gastric epithelial restitution [118], but is not essential for gastric epithelial repair [119, 120]. Interestingly, NHE1 has also been found to be involved in insulin-like growth factor II-induced proliferation and carbachol-/insulin-like growth factor II-stimulated migration of human gastric myofibroblasts [121].

Hypoxia promotes the upregulation of glycolysis to maintain ATP production, which leads to acidosis and thereby promotes the upregulation of NHEs. To date, several studies have shown that in solid tumors NHE1, as the most important cellular pH regulator, is upregulated, which can be regarded as an adaptive response of cancer cells to hypoxia and acidosis, and that the resulting intracellular alkalinization and extracellular acidification play crucial roles in cancer cell proliferation, invasion and metastasis [122, 123]. To date, various GC cell models have been established to illustrate the influence of NHE1 on the occurrence and development of GC. In a study on GC SGC-7901 cells, antisense NHE1-transfected SGC-7901 cells were found to exhibit proliferation inhibition, G1/G0 phase arrest, an increased apoptotic rate, recovery of contact inhibition and density contacts, a decreased invasive capacity, a decreased cloning efficiency in soft agar and in vivo tumorigenicity in nude mice [124]. In addition, EIPA (an inhibitor of NHE1) was found to suppress the proliferation of human GC MKN28 cells by upregulating p21 expression through the reduction of cytosolic Cl^−^ [125]. Furthermore, in human GC MKN45 and MKN74 cells, 2-aminophenoxazine-3-one (Phx-3) rapidly decreased the pHi by inhibiting NHE1, which resulted in apoptosis [126]. Notably, in human GC SGC7901 cells, NHE1 blockade decreased the pHi values, and this effect was accompanied by a significant decrease in vascular endothelial growth factor (VEGF) mRNA and protein expression [127]. The available evidence clearly shows that VEGF overexpression is critical to tumor formation and angiogenesis. Overall, the above results suggest that NHE1 exerts a strong effect on the occurrence and malignant biological behavior of GC cells. Therefore, regulating the expression and activity of NHE1 through intervention of the response to hypoxia may effectively hamper the development of GC.

### Hypoxia promotes the opening of TRPM2 channels and induces GC through autophagy

The transient receptor potential (TRP) channel superfamily belongs to the voltage-gated ion channel superfamily, which includes voltage-gated K^+^, Na^+^ and Ca^2+^ channels and related cyclic nucleotide-gated channels. These proteins form tetramers of the same subunit [128]. The proteins forming the 28 known mammalian TRP channels can be divided into six subfamilies based on amino acid sequence homology (TRPC, TRPV, TRPM, TRPA, TRPP and TRPML) [128, 129]. In many ways, the TRPM (transient receptor potential melastatin) subfamily is the largest and most diverse subfamily of TRP channels [130]. TRPM2, one of the eight members of the TRPM subfamily, is a Ca^2+^-permeable cation channel. TheTRPM2 gene is located on human chromosome 21q22.3 and consists of 32 exons encoding a protein of 1503 amino acids with a predicted molecular mass of ∼170 kDa [131, 132]. TRPM2 is mainly expressed in the central nervous system, immune cells and pancreatic cells [133] and plays a key role in immune responses [134–136], insulin secretion [137], oxidative stress [138] and body temperature control [139, 140]. In many physiological processes, TRPM2 plays a protective role. In cardiomyocytes, sustained Ca^2+^ entry through TRPM2 reduces ROS and maintains better mitochondrial bioenergetics, which protects the heart from oxidative damage after H-R [141].

A growing body of evidence indicates that TRPM2 is highly expressed in various cancers and protects the viability of many cell types after oxidative stress [142, 143]. Hypoxia can cause oxidative stress, and ADP-ribose (ADPR) produced after oxidative stress activates TRPM2 and binds to its C-terminus, which causes the channel to open. Activation of TRPM2 leads to the expression of a variety of transcription factors and kinases that are important in cell proliferation and survival, including HIF-1/2α, CREB, nuclear factor (erythroid-derived 2)-related factor-2 (Nrf2), Pyk2 and Src phosphorylation. Inhibition of the TRPM2-mediated calcium influx is associated with increased ROS production, impairments in autophagy and DNA repair, defective mitochondrial metabolism, reduced cellular bioenergetics, decreased tumor growth and increased sensitivity to chemotherapy [143]. To date, only few studies have investigated the role of TRPM2 in GC. In 2018 Almasi et al. provided the first evidence that TRPM2 is functionally expressed in GC cells and acts as a plasma membrane ion channel for calcium penetration. The authors found that shRNA-mediated downregulation of TRPM2 in two GC cell lines, AGS and MKN-45, led to a slowdown of the growth of the cells and increased the percentage of apoptotic cells. TRPM2 knockout has been found to downregulate the c-Jun terminal kinase (JNK) signaling pathway, which subsequently impairs autophagy and mitophagy. These impairments led to the accumulation of damaged mitochondria and decreases in basal and maximal mitochondrial oxygen consumption and ATP production, which ultimately caused GC cell death. In addition, the authors found that TRPM2 downregulation sensitized GC cells to paclitaxel and doxorubicin [144], which was consistent with the results of neuroblastoma studies. In a neuroblastoma study, it was found that the expression of HIF-1/2α in TRPM2-S-expressing tumor cells was significantly decreased and that the reduction in the survival of TRPM2-S-expressing cells could be rescued by functional enhancement of HIF-1α or HIF-2α. These results confirm that TRPM2 plays an important role in the regulation of ROS and HIF levels and tumor cell survival, as well as improvements in cell survival observed after doxorubicin treatment [141]. However, whether HIF exerts an effect on TRPM2 in GC remains to be investigated. Almasi et al. also found that the TRPM2 expression level is negatively correlated with the survival rate of patients with GC, which suggests that the application of TRPM2 targeting combined with chemotherapeutic drugs may be used as a strategy to improve current therapeutic effects and to improve the prognosis of patients with GC [144]. These researchers also found that TRPM2 promotes the migration, invasion and growth of GC cells through the AKT signaling pathway [145]. Based on the above-described findings, TRPM2 may promote the survival and growth of GC cells by helping them to cope with hypoxia-induced oxidative stress and by regulating hypoxia-induced transcription factor expression, mitochondrial function and mitophagy.

## Conclusions and perspectives

A hypoxic microenvironment is a key hallmark of solid tumors. Alterations and/or dysfunction of ion channels are encountered in almost all cancer types. However, one aspect that has received less attention is that the activity of ICTs is highly sensitive to a hypoxic microenvironment, highlighting a putative important role of ICTs in regulating cancer development in a such a microenvironment. Here, we outline interactive connections between multiple ICTs and various components during the hypoxic response of GC cells. We believe that both the hypoxic microenvironment and ICTs may serve as effective targets for the treatment of GC. Obtaining a better understanding of the relationship between hypoxia and ICTs in the context of GC may pave the way for a more efficacious targeted treatment of GC.

## Data Availability

Not applicable.
